# Coping with salt stress

**DOI:** 10.7554/eLife.101732

**Published:** 2024-08-30

**Authors:** Xiaoyan Liang, Caifu Jiang

**Affiliations:** 1 State Key Laboratory of Plant Environmental Resilience, College of Biological Sciences, China Agricultural University Beijing China

**Keywords:** salt stress, seed germination, seed nitrogen mobilization, *A. thaliana*

## Abstract

Salt stress delays seed germination in plants by increasing the hydrolysis of arginine-derived urea.

**Related research article** Bu Y, Dong X, Zhang R, Shen X, Liu Y, Wang S, Takano T, Liu S. 2024. Unraveling the role of urea hydrolysis in salt stress response during seed germination and seedling growth in *Arabidopsis thaliana*. *eLife* **13**:e96797. doi: 10.7554/eLife.96797.

The growth of a seed into a seedling, a process known as germination, is the first stage of the plant life cycle. Seeds contain reserves of proteins and other nutrients that must be broken down into small molecules to provide raw materials and energy for this process. One of the most important raw materials is nitrogen, which is made available through a process called nitrogen remobilization (Tan-Wilson and Wilson, 2012). However, various environmental factors, such as temperature and soil conditions, can affect the timing of germination, and many questions about the relationship between nitrogen remobilization and germination remain unanswered.

It has been proposed that salt or a plant hormone called abscisic acid can inhibit germination by blocking the breakdown or degradation of the reserves of proteins and other nutrients that are stored within the seed (Kazachkova et al., 2016; Finkelstein and Lynch, 2000; Garciarrubio et al., 1997; Penfield et al., 2006). However, experiments have shown that certain reserves can be degraded in the presence of abscisic acid (Pritchard et al., 2002). Furthermore, evidence suggests that nitrogen remobilization during seed germination enhances protein degradation and the activities of enzymes like arginase and urease, particularly under salt stress. These enzymes have key roles in the conversion of the amino acid arginine into urea and then into ammonium, which acts as a source of nitrogen (Polacco et al., 2013; Siddappa and Marathe, 2020; Bu et al., 2015; Bu et al., 2024). Now, in eLife, Yuanyuan Bu (Northeast Forestry University), Shenkui Liu (Zhejiang A&F University) and colleagues report new insights into the mechanism by which salt can inhibit germination in *Arabidopsis* seeds (Bu et al., 2024; Figure 1).

**Figure 1.**
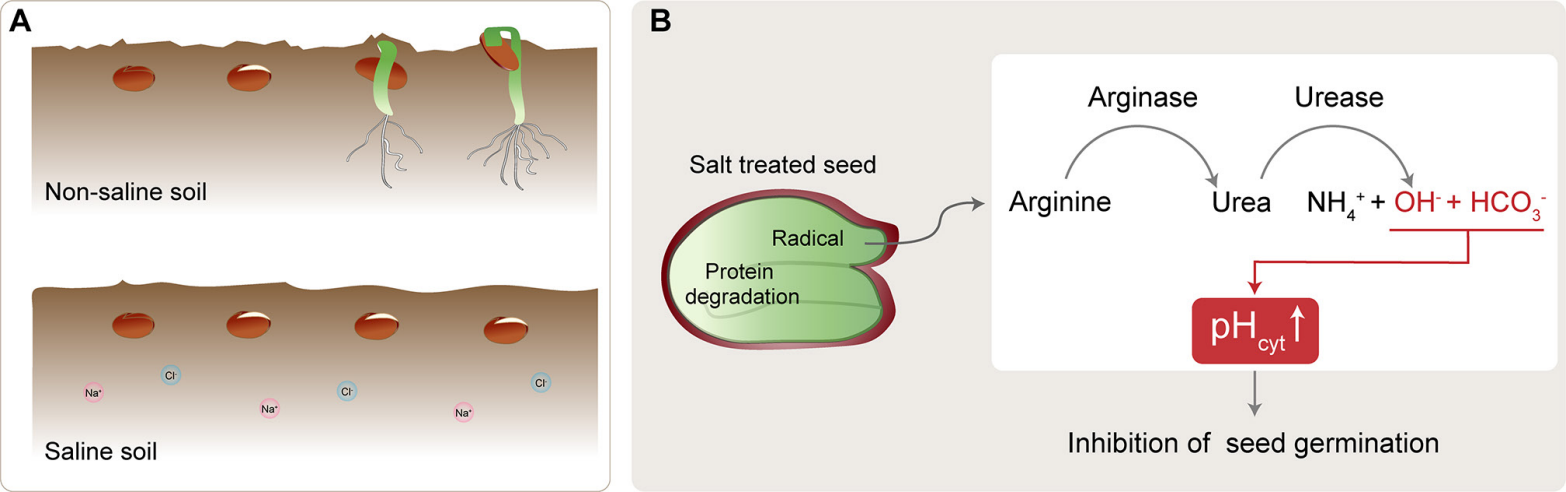
Salt-induced inhibition of seed germination. (**A**) In non-saline soil conditions (top), seeds (brown circles) germinate and grow to become seedlings (green). In saline soil (bottom), which contains more sodium (Na^+^; pink) and chlorine (Cl^-^; blue) ions, seed germination is inhibited. (**B**) Under salt stress conditions, protein degradation in the seed creates arginine, which is converted to urea by arginase. The urea is subsequently hydrolyzed by urease, resulting in increased levels of ammonium ions (NH_4_^+^), bicarbonate ions (HCO_3_^-^), and hydroxide ions (OH^-^). The bicarbonate ions and hydroxide ions increase the cytoplasmic pH (pH_cyt_), thereby inhibiting seed germination.

First, the team inhibited enzymes required for the breakdown of arginine into urea using either chemical or genetic methods. This overcame the salt-induced inhibition of germination, suggesting excessive production of urea is a key contributor to the inhibition. To test whether accumulation of the ammonium from the subsequent urea breakdown was responsible for inhibiting germination, Bu et al. added ammonium to the medium the seeds were in. However, this did not have a negative impact on seed germination, challenging the long-held belief that ammonium accumulation and toxicity are the primary causes of salt-induced inhibition of seed germination (Bu et al., 2015).

Bu et al. next considered other factors from the urea breakdown pathway that could contribute. As well as yielding ammonium, the pathway also produces hydroxide ions, which are alkaline. Measuring the cytoplasmic pH of seed cells in different conditions revealed that they are more alkaline under salt stress. Further experiments confirmed that this is due to excessive hydrolysis producing alkaline hydroxide ions (Figure 1B). Taken together, the findings demonstrate that salt stress induces excessive hydrolysis of arginine-derived urea, resulting in an increase in the cytoplasmic pH of certain cells in the seed, which in turn prevents seed germination.

The findings of Bu et al. raise the question of the evolutionary benefit of plants retaining the urea hydrolysis pathway. A possible explanation is that this pathway acts as a survival mechanism. If seeds mature, fall to the ground, and encounter salty conditions, activating urea hydrolysis and inhibiting seed germination prevents seeds growing in unfavorable conditions. If the salt concentration then decreases, following rainfall for example, germination can proceed, ensuring the species survives. This adaptation may represent a form of ‘physiological dormancy’ in seeds.

Whether seed germination is influenced by external environmental factors or internal regulatory mechanisms, it fundamentally involves the regulation of the seed’s metabolism. It remains to be seen whether other environmental stresses – such as heat, drought, heavy metals, or plant hormones – also adhere to this principle. If it turns out to be universal, the implications for growing better crops could be profound. Understanding how seeds respond to various stresses would help us to both enhance crop resilience and develop sustainable agricultural practices, which will be crucial for increasing food security in the face of climate change.
